# Professional care workforce: a rapid review of evidence supporting methods of recruitment, retention, safety, and education

**DOI:** 10.1186/s12960-023-00879-5

**Published:** 2023-12-13

**Authors:** Meg E. Morris, Natasha K. Brusco, Rachael McAleer, Stephen Billett, Lisa Brophy, Rosemary Bryant, Leeanne Carey, Amy Conley Wright, Christine East, Marion Eckert, Kristina Edvardsson, Deirdre Fetherstonhaugh, Sally Fowler-Davis, Margarita Frederico, Richard Gray, Doug McCaskie, Carol McKinstry, Rebecca Mitchell, Brian Oldenburg, Nora Shields, Karen Smith, Evelien Spelten, Nicholas Taylor, Claire Thwaites, Suzanne Young, Irene Blackberry

**Affiliations:** 1 The Victorian Rehabilitation Centre, Academic and Research Collaborative in Health (ARCH), and CERI, La Trobe University, Bundoora, VIC 3083 Australia; 2 La Trobe University, Bundoora, VIC 3086 Australia; 3 Griffith University, Mount Gravatt, QLD 4121 Australia; 4 AO Research Centre, University of South Australia, Adelaide, 5001 Australia; 5 Sydney University, Sydney, NSW 2006 Australia; 6 Rosemary Bryant AO Research Centre, University of South Australia, Adelaide, 5001 Australia; 7 Centre for Health and Care Research, Collegiate Crescent, Sheffield Hallam University, Sheffield, S1 1WB UK; 8 Alfred Health, 55 Commercial Road, Melbourne, VIC 3004 Australia; 9 Macquarie University, North Ryde, NSW 2109 Australia; 10 Silver Chain, Bourke Street, Melbourne, VIC 3000 Australia; 11 The Victorian Rehabilitation Centre and Academic and Research Collaborative in Health (ARCH) La Trobe University, Bundoora, VIC 3083 Australia; 12 CERI, La Trobe University, Bundoora, VIC 3083 Australia; 13 Florey Institute of Neuroscience and Mental Health, Heidelberg, 3084 Australia; 14 Baker Heart and Diabetes Institute, Melbourne, 3004 Australia; 15 Mercy Health, Richmond, 3121 Australia; 16 Eastern Health Academic and Research Collaborative in Health (ARCH), La Trobe University, Bundoora, 3086 Australia; 17 La Trobe Rural Health School, La Trobe University, Bendigo, 3550 Australia; 18 Rehabilitation, Ageing and Independent Living (RAIL) Research Centre, Monash University, Frankston, 3150 Australia

**Keywords:** Care economy, Workforce redesign, Workforce, Care worker, Human resources, Safety, Educational activities, Training programs, Nursing, Medicine

## Abstract

**Background:**

Across the care economy there are major shortages in the health and care workforce, as well as high rates of attrition and ill-defined career pathways. The aim of this study was to evaluate current evidence regarding methods to improve care worker recruitment, retention, safety, and education, for the professional care workforce.

**Methods:**

A rapid review of comparative interventions designed to recruit, retain, educate and care for the professional workforce in the following sectors: disability, aged care, health, mental health, family and youth services, and early childhood education and care was conducted. Embase and MEDLINE databases were searched, and studies published between January 2015 and November 2022 were included. We used the Quality Assessment tool for Quantitative Studies and the PEDro tools to evaluate study quality.

**Results:**

5594 articles were initially screened and after applying the inclusion and exclusion criteria, 30 studies were included in the rapid review. Studies most frequently reported on the professional nursing, medical and allied health workforces. Some studies focused on the single domain of care worker education (*n* = 11) while most focused on multiple domains that combined education with recruitment strategies, retention strategies or a focus on worker safety. Study quality was comparatively low with a median PEDro score of 5/10, and 77% received a weak rating on the Quality Assessment tool for Quantitative Studies. Four new workforce strategies emerged; early career rural recruitment supports rural retention; workload management is essential for workforce well-being; learning must be contextually relevant; and there is a need to differentiate recruitment, retention, and education strategies for different professional health and care workforce categories as needs vary.

**Conclusions:**

Given the critical importance of recruiting and retaining a strong health and care workforce, there is an immediate need to develop a cohesive strategy to address workforce shortfalls. This paper presents initial evidence on different interventions to address this need, and to inform care workforce recruitment and retention.

*Rapid Review registration* PROSPERO 2022 CRD42022371721 Available from: https://www.crd.york.ac.uk/prospero/display_record.php?ID=CRD42022371721

## Background

Care work refers to labour that focuses on the well-being or development of people, requiring skills in communication, interaction and evidence-based practice in healthcare and social care roles [1]. Key domains of the care economy include aged care, disability, healthcare, mental health, family care, youth services, early childhood education, indigenous services, rural health, drug and alcohol services and social housing [2]. Despite similar workforce needs and challenges faced by these sectors, individual services typically operate in silos [2] and people with multiple morbidities often present to several facilities. There is little collaboration across these industry sectors to address common problems relating to recruiting, supporting, and retaining the care workforce, to deliver high quality care. The workforce are increasingly required to innovate and improve services and adapt new technologies. Care workers also need to address the increasing divergence of consumer needs (including clients, patients family members and other informal carers) and ensure that consumers have a voice in their own care [3–5].

The COVID-19 pandemic highlighted the critical role of workers in the healthcare economy to global health security [6]. The healthcare economy is at the forefront of securing the health and well-being of citizens globally. A nation’s economy is dependent on a care workforce that is adequately resourced, supported, and remunerated [7]. The pandemic exacerbated pre-existing challenges in workforce recruitment, retention and burnout in the health and social care sectors [8–11]. There is evidence that many of these workforce issues are relevant across care economy sectors, particularly in relation to staffing levels, low staff morale and attrition [12–14].

There are several co-ordinated workforce strategies internationally (e.g., see https://www.england.nhs.uk/ournhspeople/) that have sought to establish more compassionate working environments in terms of staff well-being support and tackling discrimination. Many aim to strengthen workforce recruitment and retention through better job incentives, staff education, training and by ensuring worker safety [7]. Recruitment and retention strategies apply to the skilled, registered, and professional care workforces and the informal and unregistered workforce (care workers), in addition to volunteer and peer-support workforces.

World-wide, social care sectors have reported challenges in maintaining a professional care workforce [8–10, 15]. The care workforce has ample and growing employment opportunities, leading to high staff turnover with supply outstripping demand [3]. This increase in demand is a global trend [16] and particularly affects older care recipients in residential care settings, such as care homes. Staff burnout (a state of chronic stress and exhaustion plus chronic workplace stress that can lead to sickness and absence) is also a common, debilitating and a costly issue [17]. Career pathways across the care workforce and educational opportunities have not universally been addressed in a systematic way [6] to enable individuals to plan and sustain their contribution to professional practice.

Critical changes need to be made to foster future care economy prosperity and there is growing research literature, especially on the need to improve recruitment and retention of the care workforce. For example, the World Health Organisation (WHO) developed a guideline for increasing access to health professional workers and care workers in remote and rural areas through improved staff retention [18]. The WHO guidelines contained 17 recommendations pertaining to education, regulation, staff incentives and staff support. Sixteen recommendations had low or very low certainty of evidence, highlighting the need to develop a cohesive evidence-based strategy to address workforce shortfalls.

In addition to the WHO guidelines, a systematic review involving 34 studies and 58,188 participants evaluated interventions to assist recruitment of the professional healthcare workforce in rural and remote areas [18]. Aligned to WHO guidelines [19] for the professional care workforce, the systematic review found that optimisation of training pathways at both undergraduate and postgraduate levels was effective at improving retention. Together with other literature, there was evidence that retention was facilitated by preferential selection of university students from a rural background [20–24] and supporting rurally placed health professionals to take further education and training [25–29]. A narrative review by Beccaria et al. (2022) [30] showed the importance of attachment to place in retaining a sustainable care economy workforce. Rapid reviews by Moriarty (2019) [31] and Marafu (2019) [32] also highlighted the value of continuous professional development in workforce retention yet these were limited to the health sector.

With previous reviews limited to pre-COVID-19 pandemic literature, the aim of this rapid review was to present recent evidence (January 2015–November 2022) across care economy sectors, settings, and geographical regions to establish evidence-based strategies to improve professional workforce recruitment, retention, safety, and education. We also aimed to examine whether new approaches were aligned with the WHO guidelines on health workforce development, attraction, recruitment, and retention in rural and remote areas [19]. Our synthesis also provides a critical appraisal of opportunities for learning and improvement across care sectors to facilitate the adoption of effective cross-sector interventions and policies.

## Methods

The rapid review, focussed on the professional care workforce, was prospectively registered with PROSPERO (PROSPERO 2022 CRD42022371721 https://www.crd.york.ac.uk/prospero/display_record.php?ID=CRD42022371721) and PRISMA [33, 34]. The approach was based on methods of Murad et al. (2017), who suggested how review results can be synthesised and the certainty of evidence estimated when a meta-analysis cannot be completed. Defining the care workforce can be a challenge [35]. For example, the line between direct and indirect care is often not made clear, where workers such as cleaners and chefs play an important but indirect role in care. In addition, for people receiving support to live in the community, unpaid care plays a crucial and often under-acknowledged role [10]. For this review, we examined the professional care workforce, defined as paid, educated, skilled workers providing direct care in home, community, hospital, residential aged care and other social service settings. We did not examine the literature on personal care assistants, nursing assistants or allied health assistants. The research question for the review was, what methods can improve the recruitment, retention, safety, and education of the professional care workforce?

### Search strategy

This rapid review was conducted in Embase and MEDLINE. The search was be limited to studies published in English, and time limited to between January 2015 and November 2022 (refer to Appendix 1 for the full MEDLINE search strategy).

### Inclusion and exclusion criteria

Inclusion criteria:

1. “Consumer focused” care workforce professions or professionals, inclusive of but not limited to people paid to work in healthcare services, aged care, home care, community care, disability, rehabilitation, social housing or homelessness, early childhood education and care and child protection, drug and alcohol services, rural and remote care, mental health, family services, domestic violence or Indigenous health and social care.
2. Interventions pertaining to recruitment, retention, safety, and education of the “client focused” professional care workforce.
3. All forms of quantitative research with adequate data and information provided to ascertain results.
4. Must include a comparator (pre–post, RCT against different interventions).
5. English language articles.
6. Studies published between the months of January 2015–November 2022.

Exclusion criteria:

1. Professions or staff other than the direct “client focused” care workforce, as defined above.
2. Unqualified, non-professional, unskilled or non-registered care workforce
3. Peer support workers
4. Pre-implementation, pilot, and feasibility studies of an intervention.
5. Qualitative studies, opinion pieces, commentaries, editorials, and theses.
6. Articles published prior to the year 2015.

### Participants

The care workforce as defined in the inclusion criteria. This rapid review was focused on the ***qualified professional care workforce***, and any patient, client, or consumer outcomes were not reported.

### Interventions

Interventions involved the care workforce and related to staff recruitment, retention, safety, and education. Retention pertains to the longevity of a period of employment within the care workforce. Recruitment refers to the ability to fill vacant advertised positions. Safety pertains to all elements of working safely from the care workforce perspective. Examples pertain to occupational health and safety and include, for example, needle stick injuries, workplace violence, back injuries, burnout. Education is the ongoing education, training, and professional development of the care workforce. Included studies were required to have data and a comparator, for example, pre and post intervention data. Studies were excluded if they only described the pre-implementation phase of an intervention, or if they were a pilot or feasibility study, case report or descriptive summary.

### Outcome

The primary outcome was interventions, policies and procedures designed to support, retain, and facilitate the professional, qualified care workforce and synthesise of the evidence from these outcomes.

### Data extraction

Data from database searches were downloaded into Endnote, duplicates removed, then exported to Covidence. As per Rapid Review guidelines [36], two researchers conducted a pilot screening exercise using the same 30–50 abstracts to calibrate and test the review criteria, with discrepancies resolved by discussion and a review of the full text as required. One researcher then screened the titles and abstracts of all identified studies against the inclusion and exclusion criteria. Two researchers then conducted a second pilot exercise to review the full text articles, using the same 5–10 full text articles to ensure consistency, with discrepancies resolved by discussion. A researcher then reviewed the full texts of the articles to determine the final selection. The final selected articles also had their reference lists hand searched for any additional articles of interest.

Data extraction of full text included articles was completed by one reviewer and a second reviewer checked for correctness and completeness of extracted data, with discrepancies resolved by discussion. The Physiotherapy Evidence Database (PEDro) scale [37, 38] and the Quality Assessment Tool for Quantitative Studies (QATQS) [39] were used to analyse the quality of the included articles. We used PEDro, because it is a validated tool for objectively measuring the reliability and clinical usefulness of trials. The Canadian QATQS added more detail to the quality of public health investigations. Data extraction included details of the intervention (abbreviated TIDIER checklist), study characteristics, control group—population and primary outcome results, intervention quality scores.

### Risk of bias and quality assessment

The PEDro scale [37, 38] items include: eligibility criteria specified; random allocation of subjects to groups; allocation concealment; similarity of groups at baseline regarding the most important prognostic indicators; blinding of all subjects; blinding of all therapists who administered the therapy or intervention; blinding of all assessors who measured at least one key outcome; measures of at least one key outcome were obtained from more than 85% of the subjects initially allocated to groups; all subjects for whom outcome measures were available received the treatment or control condition as allocated or, where this was not the case, data for at least one key outcome was analysed by “intention to treat”; the results of between-group statistical comparisons are reported for at least one key outcome, and the study provides both point measures and measures of variability for at least one key outcome.

The Quality Assessment Tool for Quantitative Studies rated the methodological quality for each study based on selection bias, study design, confounders, blinding, data collection methods, withdrawals and dropouts, intervention integrity and the analysis [39]. This assessment tool provides an overall rating of weak, medium, or high quality.

### Data synthesis and analysis

A purpose-built Excel database was used to extract study characteristics, intervention details, outcome measures and risk of bias. A descriptive analysis was provided for interventions for each of the different care workforces, in addition a descriptive analysis was provided for each of the four intervention types. Meta-analysis was planned when two or more studies had heterogeneity with the following factors: discipline of the care workforce, the type of intervention, primary outcome of the intervention, and comparable follow-up period. Data for synthesis included primary outcomes which measure the intervention impact on care workforce recruitment, retention, safety, or education. When two or more studies met these criteria, Review Manager (RevMan) Version 5.4. was used to complete the meta-analysis based on the mean-difference and measures of variability.

## Results

**Fig. 1:**
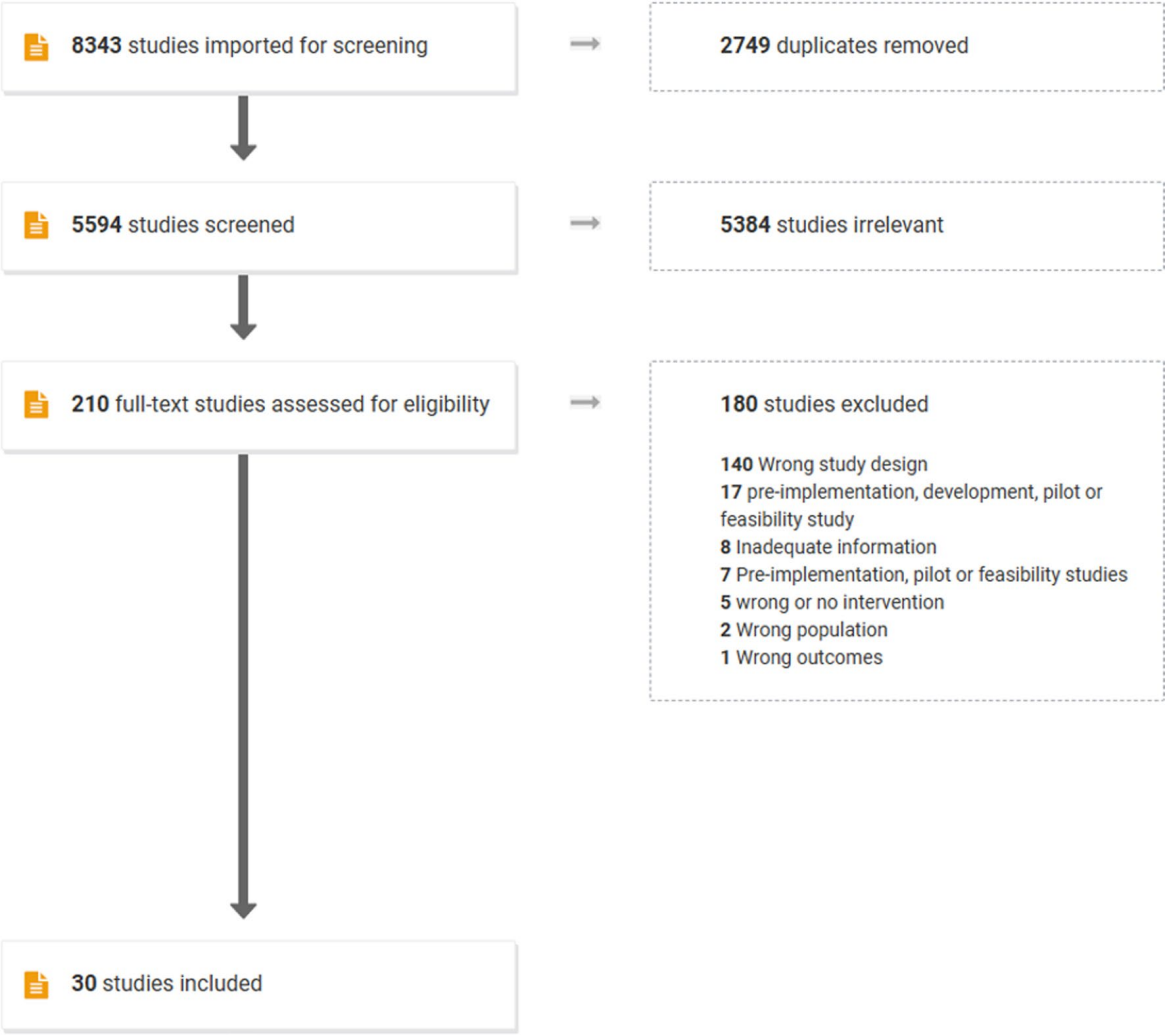
PRISMA flow chart

The initial search strategy resulted in 8343 studies, of which 2749 were duplicates. Following screening of title and abstracts, as well as full text, 30 studies were included [40–69] (Fig. 1).

**Fig. 2:**
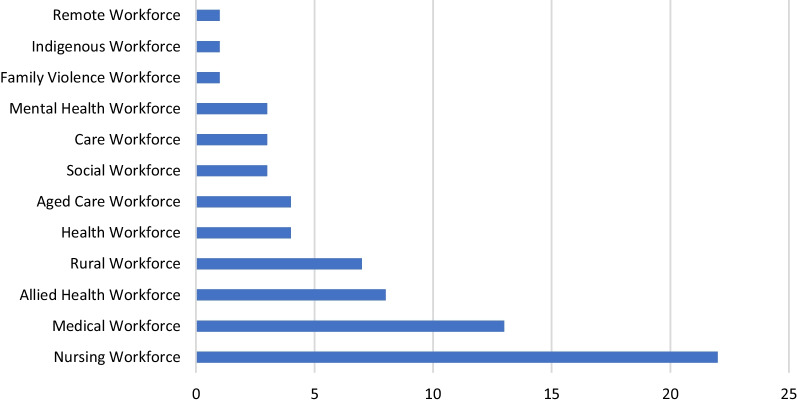
Number of studies reporting on the different members of the ‘professional’ care workforce

Study characteristics and results are detailed in Tables 1 and 2, respectively, noting that studies could report on more than one workforce intervention category. Studies most frequently reported on the nursing (*n* = 22), medical (*n* = 13) and allied health (*n* = 8) workforces (Fig. 2). Regarding the domains the intervention aimed to influence, some studies focused on the single domain of education (*n* = 11) [50, 51, 55, 57, 58, 62, 64] while most focused on multiple domains which combined education with recruitment, retention and/or safety. Seven studies included consumer engagement or co-design, as defined by McKercher et al. (2022) [5], while none of the studies included an economic evaluation. Meta-analysis was not appropriate due to the heterogeneity of the intervention, the included workforce, the setting, the study design, and the outcome measures.

**Table 1:** Characteristics of the included studies

Included papers		Intervention (Abbreviated TIDIER Checklist)							Study characteristics							
No	Citation	NAME? Intervention Name	WHY? Intervention aim	WHAT? Intervention/methods	WHAT? CODED: Intervention/methods	WHO? Intervention provider	HOW? The intervention provided	Domains the intervention aims to influence	Workforce	Country (and country income*	Setting	Study design	Economic evaluation?	Consumer engagement?	Primary outcome measure	Domain for the primary outcome
1	Abdulla et al. 2020 [40]	Not stated	Improve knowledge and skill acquisition	Primary Health Care nurses’ knowledge before and after immunization education program	Inservice training/education program	Primary Health Care Centres’ (PHCCs) work force training department (WFTD)	Immunisation course (no details provided); and practical (minimum of 10 vaccinations)	Education	Nursing Workforce	Qatar (high-income)	Primary Health (health centres, school health, home healthcare, community health)	Mixed method (pre–post-survey/interviews)	No	No	Knowledge (administration of vaccinations)	Education
2	Alwy Al-Beity et al. 2020 [41]	Helping Mothers Survive Bleeding after Birth (HMS BAB)	Improve knowledge and skill acquisition (decrease mortality rates)	HMS BAB training is competency-based simulation training for all health workers in a maternity unit	Inservice training/education program	HMS BAB curriculum developed by Laerdal Global Health and Jhpiego (part of larger HMS BAB trial)	Master trainers trained 12 district trainers (1 week). Health workers = 1 day of training. 2 workers chosen to be ‘peer practice facilitators’ trained to lead 8 weekly mandatory clinical scenario practice drills (30–40 min)	Education	Nursing, Medical and Health Workforce	East Africa (Tanzania; lower-middle income)	Rural or semi-rural health facilities *n* = 61	Pre–post survey	No	No	Knowledge (pre- and post-training assessment) Skill acquisition (3 simulated scenarios)	Education
3	Ayisi-Boateng et al. 2022 [42]	Not stated	Improve knowledge	Alzheimer’s disease and related dementias (ADRDs) workshop	Inservice training/education program	Facilitators from family medicine, neurology, geriatrics, psychiatry and public health	4-h in-person educational content/workshop on ADRD	Education	Medical, Nursing, Allied Health and Aged Care Workforce	Ghana (lower-middle income)	Public and private healthcare facilities in Kumasi, Ghana	Pre–post survey	No	No	Knowledge Alzheimer’s Disease Knowledge Scale	Education
4	Azoulay et al. 2021 [43]	Dedicated Liver Surgery Program	Improve knowledge and skill acquisition; improve staff retention/recruitment	Senior surgeon teaching liver surgery to junior colleagues, as well as mentor and develop their academic production	Formal education program	Chaim Sheba Medical Centre (Israel)	4 week resident rotations; WhatsApp group to share education; conferences; senior surgeon mentoring; senior liver specialist anaesthetist meetings; morbidity and mortality conference	Recruitment, Retention and Education	Medical Workforce	Israel (high income)	University-affiliated hospital	Pre–post survey	No	No	Surgery output, publication output, education uptake (WhatsApp educational group)	Education
5	Bennett et al. 2022 [44]	Not stated	Improve knowledge and skill acquisition	Training aged care workforce compassion and person centred care by completing activities wearing the aged simulation training suit (ASTS)	Inservice training/education program	ACH Group	3 h training activity using an ASTS. Staff completed functional daily tasks for approximately 30 min while wearing the suit	Education	Aged Care and Nursing Workforce	Australia (high income)	Residential care facilities	Pre–post survey	No	No	Knowledge and skill acquisition The Compassion Competence Scale (CCS) and Person-centred Care Assessment Tool (P-CAT)	Education
6	Chicoine 2022 [45]	ECHO-CD (Extension for Community Healthcare Outcomes—Concurrent Disorders)	Improve self-efficacy (knowledge and skill acquisition)	ECHO is a videoconference-based, interprofessional tele-mentoring model to support and build capacity in CD healthcare professionals	Inservice training/education program	Not stated in this publication	Pairing healthcare professionals (the “Spokes”), with an interdisciplinary team of experts (the “Hub”) at a centralized academic centre. 90-min online educational sessions every 2 weeks for 20 sessions	Recruitment and Education	Nursing and Mental Health Workforce	Canada (high income)	Hospital, community and primary care	Pre–post survey (baseline, 6 months and 12 months)	No	No	Self-efficacy	Education
7	Clancy et al. 2020 [46]	‘My Early Relational Trauma Informed Learning’ (MERTIL) program	Improve knowledge and skill acquisition	MERTIL is an online learning and face-to-face workshop-based trauma-informed training for MCH (maternal child health) nurses	Inservice training/education program	Victorian State Government	20-h program of online learning (13 h) and face-to-face clinical skills workshops (7 h)	Retention and Education	Nursing, Allied Health, Family Violence and Social Workforce	Australia (high income)	MCH staff across the state	Pre–post survey	No	No	Competency and knowledge	Education
8	Dierkes et al. 2022 [47]	State-level staffing mandate	Improve staff retention	Introduction of staff mandates to improve patient safety	Policy/protocol change	Not stated	Health services introduced nursing staff ratio mandates in California hospitals	Safety, Recruitment and Retention	Nursing Workforce	USA (high income)	Hospitals	Longitudinal, pre-test/post-test design	No	No	Nurse staffing levels defined as registered nurse hours per patient day (HPPD)	Safety
9	Downing et al. 2016 [48]	Link-nurse programme	Improve knowledge and skill acquisition; improve service provision	Link-nurse training and mentorship to equip nurses from different wards with knowledge/skills to provide generalist palliative care alongside their clinical team	Inservice training/education program	Mulago Hospital	5 day program = 3 days training, followed by mentorship/support supervision onwards, and 2 day training 3 months later	Recruitment, Retention and Education	Nursing Workforce	East Africa (Uganda; low income)	Hospital	Mixed methods—pre-test/post-test design	No	No	Confidence/competence	Education
10	Gajewski et al. 2019 [49]	ML (medical licentiates) training programme	Improve knowledge and skill acquisition; improve service provision	Task-shifting by training non-physician clinicians (NPCs) called medical licentiates to perform common surgeries in rural hospitals due to surgeon shortages	Inservice training/education program	Clinical Officer Surgical Training in Africa (COST-Africa)	Program designed to enhance surgical skills of MLs—3 month intensive surgery course; 3 monthly supervision by specialist surgeons once deployed	Education, Recruitment, Retention and Safety	Medical and Rural Workforce	Africa (Zambia; low income)	Hospital	RCT—matched-pairs	No	No	Outputs Number of selected common general surgical interventions—measured with extended theatre register	Education
11	Gordon et al. 2022 [50]	reBoot Camp	Improve knowledge; improve technological skill acquisition	reBoot Camp training program was created as ongoing education. Identified need by physicians to facilitate electronic health record (EHR) implementation	Inservice training/education program	Mayo Clinic—Clinical Systems Education	reBoot Camp is an intensive and interactive refresher course consisting of 2-day sessions on EHR topics relevant to ambulatory care	Education	Medical Workforce	USA (high income)	Hospital	Pre-test/post-test design	No	Yes	Knowledge Proficiency Score	Education
12	Islam et al. 2020 [51]	Four Steps to Building Dementia Practice in Primary Care	Improve knowledge and skill acquisition: to lead practice change	Free training program developed on the ‘Four Steps to Building Dementia Practice in Primary Care’ on the timely diagnosis and management of dementia	Inservice training/education program	Not stated	Face-to-face (3.5 h) or online (4 × 1 h modules) training program	Education	Nursing Workforce	Australia (high income)	Primary health care facilities	longitudinal study—pre-test/post-test design	No	Yes	Knowledge Self-perceived levels of importance, knowledge and confidence	Education
13	Jafari et al. 2020 [52]	ECHO-Chicago Geriatrics (Extension for Community Healthcare Outcomes)	Improve knowledge and skill acquisition; additional mentoring	ECHO connects academic medical specialists and community health care providers via videoconferencing for free education sessions	Inservice training/education program	University of Chicago	12 sessions × 1 h of didactic lecture (20–30 min) and telemonitoring case study discussions completed via videoconferencing technology facilitated by × 1 UCM geriatrician, a geriatric nurse educator and at least one geriatric social worker	Retention and Education	Medical, Care and Aged Care Workforce	USA (high income)	Hospitals, aged care facilities, community clinics	Pre-test/post-test design	No	No	Knowledge Self-efficacy Behaviour change Frequency of practice behaviours	Education
14	Jedwab et al. 2022 [53]	Electronic medical record (EMR) system implementation	Improve staff retention; improve staff safety	EMR system implementation across 6 hospitals of a large tertiary healthcare organisation	Impact of technology implementation	Not stated	Surveys collected pre-electronic medical record implementation prior to COVID pandemic and 18-month post implementation during the pandemic	Retention, Safety and Education	Nursing Workforce	Australia (high income)	Hospitals-only inpatient staff	Cross sectional pre-test/post-test design	No	Yes	Well-being; Work engagement; Motivation to use technology; Experience using technology	Safety (well-being)
15	Johnston et al. 2020 [54]	The Training for Health Equity Network (THEnet)	Improve staff recruitment; Improve knowledge and skill acquisition; Improve health service provision	THEnet Graduate Outcome Study (GOS) of medical graduates	Formal education program	The Training for Health Equity Network (THEnet)	THEnet is a community-of-practice of 13 health professional education institutions with a focus on delivering socially accountable education to produce a fit-for-purpose medical workforce	Recruitment, Retention and Education	Rural and Medical Workforce	Australia (high income), The Philippines (lower-middle income), Sudan (low income), South Africa (upper-middle income), Nepal (lower-middle income), Canada (high income)	University	Mixed methods, pre-test/post-test design	No	No	Recruitment Intention to practice in rural and other underserved areas	Recruitment
16	Martin et al. 2019 [55]	Quitskills (part of the Tackling Indigenous Smoking Program)	Improve knowledge and skill acquisition; Improve cultural competency	Quitskills training program for health professionals working with First Nations people who smoke	Inservice training/education program	Cancer Council South Australia—Australian Government	3 day course aimed at being culturally relevant to increase health professionals’ skills, knowledge and confidence to assess and discuss smoking behaviour and support First Nations people to quit smoking	Education	Health, Indigenous and Rural Workforce	Australia (high income)	Healthcare facilities	Pre-test/post-test design	No	Yes	Knowledge and confidence	Education
17	Mikolajczyk et al. 2021 [56]	Inpatient hepatology resident curriculum	Improve knowledge and skill acquisition	A mandatory, inpatient, hepatology resident curriculum	Formal education program	University of Chicago Medical centre	Mandatory hepatology rotation lasts for 2 weeks. 15 core topics, patient care, weekly conferences, literature review, didactic sessions, online educational activities	Recruitment and Education	Medical Workforce	USA (high income)	Hospital	Pre-test/post-test design	No	No	Knowledge 12 MC questions from the Medical Knowledge Self-Assessment Program	Education
18	Morshed et al. 2017 [57]	Evidence-based cancer control (EBCC) training	Improve knowledge and skill acquisition	Online evidence-based cancer control (EBCC) training to increase evidence-based interventions to control obesity and cancer via exercise/diet	Inservice training/education program	Prevention Research Centre (St Louis)	Online EBCC training of 26 skills for EBCC care over 6 modules Interactions practiced via simulations	Education	Care, Nursing and Allied Health Workforce	USA (high income)	Public health settings (not specific)	Pre-test/post-test design	No	No	Knowledge Skill-based competence	Education
19	Murthy et al. 2020 [58]	Not stated	Improve knowledge and skill acquisition	Determine if similar skill acquisition with low fidelity (LF) vs high fidelity (HF) simulation training course in clinical breast exam (CBE)	Inservice training/education program	University Teaching Hospital and the Centre for Surgery and Public Health Brigham and Women’s Hospital in Boston	1-day CBE simulation training course. Practice with trainer	Education	Medical Workforce	East Africa (Rwanda; low income)	Hospital	Single centre randomised cross over—pre-test/post-test design	No	No	Knowledge	Education
20	Neikrug et al. 2022 [59]	UCI Train New Trainers (TNT) Primary Care Psychiatry Fellowship	Improve knowledge and skill acquisition	Fellowship program for professionals working in primary care-based psychiatric care	Formal education program	University of California Irvine and University of California Davis	1 year fellowship for behavioural health workforce primary care providers	Safety and Education	Mental Health, Medical and Nursing Workforce	USA (high income)	Primary care	Pre-test/post-test design	No	No	Knowledge 50 item unvalidated MC exam developed by researchers	Education
21	Ortega et al. 2018 [60]	Nursing Leadership: Empowering Nurses in Latin America and the Caribbean	Improve knowledge and skill acquisition	Online nursing leadership course in English and Spanish for nurses in leadership roles	Inservice training/education program	Pan American Health Organization (PAHO) Virtual Campus	Asynchronous, online 8 module courses. Expected to complete within 3 months. 3 cohorts—Cohort 1 (English speaking), Cohort 2 (Spanish speaking), Cohort 3 (in Uruguay)	Retention and Education	Nursing Workforce	USA (high income)	Public facilities	Retrospective pre-test/post-test design	No	No	Knowledge Eight pre–post within-module exams	Education
22	Ortega et al. 2021 [61]	Not stated	Improve knowledge and skill acquisition	Program to improve the integrated response of mental health crisis teams through simulated patient training	Inservice training/education program	Maudsley Simulation	Program of 5 interprofessional simulation courses (11 sessions) focusing on core skills to improve mental health crisis situations. Simulation scenarios with trained actors of 10–15 min with 30–40 min debrief	Safety and Education	Mental Health, Nursing, Allied Health, Social and Medical Workforce	UK (high income)	All clinical facilities	Mixed methods—pre-test/post-test design	No	No	Measure of social and cognitive abilities in demanding situations The Human Factors Skills for Healthcare Instrument (HuFSHI)	Education
23	Parmar et al. 2022 [62]	The Foundational Caregiver-Centered Care Education program	Improve knowledge and skill acquisition	Person-centred competency-based education program for the care workforce working with family caregivers	Inservice training/education program	University of Alberta ethics	1 h free competency education to identify, assess, support and partner with family caregivers. 6 modules that follow the domains in the Caregiver-Centered Care Competency Framework	Education	Allied Health, Nursing, Aged Care, Social and Medical Workforce	USA (high income)	Primary care, hospital, home care, aged care	Mixed methods—pre-test/post-test design	No	Yes	Knowledge and confidence Caregiver-Centered Care Knowledge Assessment Test	Education
24	Playford et al. 2020 [63]	Not stated (rural/remote clinical placement)	Improve rural recruitment/retention	Rural/remote clinical placement in final year of study. Followed up at 1 year and 15–17 year post-graduation to determine location of practice (rural/urban)	Formal education program	University of Western Australia—University Department of Rural Health (UDRH)	Nursing and allied health students enrolled at an urban campus and completed a rural placement of 2–18 weeks in final year of course	Recruitment, Retention and Education	Allied Health, Nursing, Rural and Remote Workforce	Australia (high income)	Western Australian UDRH	Longitudinal cohort study—pre-test/post-test design	No	No	Recruitment and retention to rural workplace Location of practice	Recruitment
25	Risendal et al. 2022 [64]	iSURVIVE	Improve knowledge and skill acquisition	Cancer survivorship education program for rural primary care practice health professionals	Inservice training/education program	High Plains Research Network at Department of Family Medicine University of Colorado	Multimodal curriculum using SOuND Team Training (Trademark)—4 × 1 h face-to-face full practice team sessions. Didactic and interactive. Supplemental series × 12 monthly 1 h interactive webinars	Education	Rural, Health, Care, Nursing, and Allied Health Workforce	USA (high income)	Primary care	Mixed methods—pre-test/post-test design	No	Yes	Knowledge 14 item questionnaire	Education
26	Salehi et al. 2021 [65]	Not stated	Improve knowledge and skill acquisition; Improve staff recruitment	National paediatric nurse training program evaluation after 4 years of graduates	Formal education program	SickKids-Ghana Paediatric Nursing Education Partnership; Ghana College of Nurses and Midwives	1 year competency-based training program; lectures, case-based learning, simulation, extensive clinical practicum. Content = family centred care, strengths-based nursing and gender equality, primary care, managing acute and challenging hospital patients, emergency care, leadership development	Recruitment, Retention and Education	Nursing Workforce	West Africa (Ghana; lower-middle income)	Training centres × 3 across the country; hospitals	Mixed method—pre-test/post-test design	No	Yes	Knowledge, confidence and clinical skills	Education
27	Sibrian et al. 2022 [66]	Not stated	Improve knowledge and skill acquisition	Virtual education approach to address learning needs during COVID	New graduate clinical nurses (NGCNs) virtual program due to remote working and social distancing	Not stated	10 week online—active learning strategies, including unfolding clinical case studies, self-reflection, small group discussion, role playing, debriefing. Wellness/stress management topics. Online preceptorship and 6 virtual education sessions	Retention, Safety and Education	Nursing Workforce	USA (high income)	Healthcare facilities with new graduate programs	Pre-test/post-test design	No	No	Knowledge Casey-Fink Graduate Nurse Experience survey (revised)	Education
28	Tran et al. 2019 [67]	APN Leadership Development Program	Improve knowledge and skill acquisition	Leadership and management fellowship for advanced practice nurses (APNs)—certified nurse midwives (CNMs) and nurse practitioners (NPs)	Formal education program	Not stated	1 year fellowship—3 intensive face-to-face leadership retreats; 2 monthly distance-based learning activities. Distance and online workshops, seminars, team learning	Retention, Safety and Education	Nursing and Rural Workforce	USA (high income)	Academic health centres; private practice	Pre-test/post-test design	No	No	Knowledge Leadership/management competencies	Education
29	Vesel et al. 2015 [68]	Helping Health Workers Cope (HHWC) project	Improve coping skills/stress levels and relationships	HHWC offers counselling and psychological training on coping, stress and provider–provider and provider–client relationships	Inservice training/education program	Not stated	Individual intake counselling assessment. 10 × group counselling sessions. Health workers grouped into women and men’s groups and met weekly. Trained on stress management, self-care and client-care. Refresher training after 9 months	Retention, Safety and Education	Rural, Health and Nursing Workforce	West Africa (Sierra Leone; low income)	Primary health care facilities	Retrospective pre-test and a post survey	No	No	Safety (mental health) Coping skills, perceived stress levels and changes in relationships (pre to post)	Safety (well-being)
30	Zhang et al. 2021 [69]	‘appointment–triage–disinfection’ work pattern	Improve staff safety (mental health; workload; stress)	The work pattern (triage protocol and disinfection frequency) of the hospital pre-COVID and post-COVID outbreak; quarterly mental health surveys to assess the mental status of the military healthcare providers	Policy/protocol change	United Nations	Change to work pattern due to COVID pandemic. Patients must make appointment, triaged by specialist based on risk (temperature taken), patient to fever or routine clinic. Disinfect hospital—high risk areas × 2/day, lower risk × 1/day	Retention and Safety	Medical, Nursing and Allied Health Workforce	South Sudan (low income)	United Nations peacekeeping field hospital	Mixed methods (Pre–post surveys)	No	No	Safety (mental health) Perceived Stress Scale and generalised Anxiety Disorder before/after the COVID outbreak	Safety (well-being)

*Classification based on the World Bank Country and Lending Groups—World Bank Data Help Desk)

**Table 2:** Results of the included studies

Included papers		Population and outcomes Control group (or pre-intervention)				Population and outcomes Intervention group (or post-intervention)					
No	Citation	Number	Age	Gender (M = Male; F = Female; O = Other)	Primary outcome measure result	Number	Age	Gender (M = Male; F = Female; O = Other)	Primary outcome measure result	Mean difference between groups Primary outcome measure result (intervention minus Control)	Mean difference between groups
1	Abdulla et al. 2020 [40]	*n* = 64 cohort 1 pre and post test	25–33 = 40 34–44 = 21 45–54 = 3	M = 4 F = 60	Knowledge Percentages presented for each question	*n* = 56 cohort 2 same pre and post-test as cohort 1	25–33 = 28 34–44 = 22 45–54 = 6	M = 3 F = 53	Knowledge Percentages presented for each question	Pre/post results presented for Cohort 1 and 2, but not between cohorts	Significant Diff
2	Alwy Al-Beity et al. 2020 [41]	*n* = 636	Not stated	Not stated	Knowledge—74.2 mean at pre-training, 89.2 mean immediately after the training. Skill acquisition—increased from 38.2 mean to 85.4 immediately post-training	*n* = 193 (10-month assessment in a subset of health workers)	Not stated	Not stated	Knowledge—85.4 mean at the 10-month follow-up. Skill acquisition—significant overall decline of skills at 10-month follow-up from 85.4 mean to 80.8	Knowledge: overall scores increased from 78 to 93% (*p* < 0.0002). Skill acquisition: scores increased from 38 to 83% (*p* < 0.000)	Significant Diff
3	Ayisi-Boateng et al. 2022 [42]	*n* = 49	Mean 34.6 (± 6.82)—range 22–50 years	M = 24 F = 25	19.8 (± 4.3) out of 30 Score relates to participants’ knowledge	*n* = 49	mean 34.6 (± 6.82)—range 22–50 years	M = 24 F = 25	23.2 (± 4.0) out of 30 Score relates to participants’ knowledge	increase in the proportion of participants who had correct answers in all the seven domains (*p* < 0.01)	Significant Diff
4	Azoulay et al. 2021 [43]	Resident numbers not provided	Not stated	not stated	Number of surgeries: 2/138 (0.1%) hepatectomies during period 1 (first 2 years). No WhatsApp HPB Group in period 1. Conferences = no formal conference/presentations in period 1. Publications = 2	Resident numbers not provided	Not stated	Not stated	Number of surgeries: of 81/188 (43.1%) hepatectomies during period 2. WhatsApp HPB Group in period 2 very active. Conferences = 11. Presentations = 7. Publications = 12	Number of surgeries (representing increase in knowledge): 40-fold increase. Publications/presentations = sixfold increase	Significant Diff
5	Bennett et al. 2022 [44]	*n* = 160	Not stated	Not stated	CCS Total score (median) = 81 (range 18–90) P-CAT Total Score (median) = 43 (range 13–65)	*n* = 160	Not stated	Not stated	CCS Total score: media*n* = 85 (range 63–90) P-CAT Total Score: media*n* = 44 (range 29–57)	Difference in CCS Total score = *p* = 0.007; P-CAT Total Score = *P* ≤ 0.001	Significant Diff
6	Chicoine 2022 [45]	*n* = 28	39.1 mean	M = 1 F = 27	Self-efficacy = 7.8 (least square means)	*n* = 19 (6 month) *n* = 12 (12 month)	Not Stated	Not Stated	Self-efficacy—6 months = 7.8; 12 months = 7.9	significant changes in self-efficacy at 12-month follow-up (*P* = 0.0213), among the nurses who attended more than 25% of the 20-session curriculum	Significant Diff
7	Clancy et al. 2020 [46]	*n* = 1450	64% aged 51 + years	M = 0 F = 1450	Competency/knowledge Participants reported low to moderate confidence in their ability to recognize or respond to signs/symptoms of early relational trauma in families	*n* = 734 (post) *n* = 651 (follow-up at 2–3 months post)	Not stated	M = 0 F = 734 (post) F = 651 (follow-up)	Competency/knowledge Increases in confidence and capability	Competency/knowledge Increases in confidence and capability (*p* < 0.01)	Significant Diff
8	Dierkes et al. 2022 [47]	Not stated	Not stated	Not stated	HPPD pre-mandate (mean): California = 6.03; other States = 6.03	Not stated	Not stated	Not stated	HPPD pre-mandate (mean): California = 7.90; other States 6.73	Not reported; states *p* < 0.05	Significant Diff
9	Downing et al. 2016 [48]	*n* = 27	Not stated	M = 1 F = 26	Confidence/competence = least confident in morphine prescribing (mean = 2.32), models of palliative care (mean = 2.48), end-of-life care (mean = 2.68) and bereavement support (mean = 2.76)	*n* = 25	Not stated	Unclear	Confidence/competence = least confident in morphine prescribing (mean = 2.32), models of palliative care (mean = 2.48), end-of-life care (mean = 2.68) and bereavement support (mean = 2.76)	Confidence/competence = *p* < 0.001 for what is palliative care, concept of total pain, models of palliative care provision, basic communication, bereavement support, pain assessment and management, Morphine prescribing, end of life care, caring for children	Significant Diff
10	Gajewski et al. 2019 [49]	*n* = 8	Not stated	Not stated	Knowledge demonstrated through increase surgeries; caesarean sections pre results: = 990; post results: 525 (− 47% change); Common surgeries pre results: = 417; post results: 437 (+ 4.8% change)	*n* = 9	Not stated	Not stated	Knowledge demonstrated through increase surgeries; caesarean sections pre results: = 900; post results: 1037 (15.2% change); Common surgeries pre results: = 508; post results: 483 (− 4.9% change)	In 5 pairs intervention hospitals performed more caesarean sections (*p* = 0.015)	Significant Diff
11	Gordon et al. 2022 [50]	General navigation *n* = 163; Documentation *n* = 158; Order entry = 150; Medications = 150; In-basket = 163; Reports = 132	Not stated	Not stated	PS (baseline mean scores) General navigation 58.5; Documentation 57.7; Order entry 56,4; Medications 57.4; In-basket 52.3; Reports 24.1	General navigation *n* = 163; Documentation *n* = 158; Order entry = 150; Medications = 150; In-basket = 163; Reports = 132	Not stated	Not stated	PS (1 month post mean scores) General navigation 72.0; Documentation 70.4; Order entry = 71.0; Medications = 70.1; In-basket = 70.5; Reports = 34.2	Before to after reBoot camp: *p* < 0.001 for all domains; Sustained at 6 months for all domains	Significant Diff
12	Islam et al. 2020 [51]	*n* = 1290 (*n* = 471 face to face; *n* = 819 step 1 online*) * *n* = 443 at step 2; *n* = 307 step 3; *n* = 253 step 4	Not stated	Not stated	1. Self-perceived levels of importance, knowledge and confidence = individual statistics not provided (in graph format). 2. 4 step assessments (average): Step 1 = 0.93; step 2 = 1.20; step 3 = 1.09; step 4 = 0.78	Post: *n* = 471 face-to-face 6 months follow-up: unclear	Not stated	Not stated	1. Self-perceived levels of importance, knowledge and confidence: improvement in all areas; 2. 4 step assessments at follow-up only (average): Step 1 = 2.27; step 2 = 2.42; step 3 = 2.36; step 4 = 2.15	1. Self-perceived levels of importance, knowledge and confidence = differences between pre and post scores significant all 3 variables; step 1 = 1.34 *p* < 0.01; step 2 = 1.21 *p* < 0.01; step 3 = 1.26 *p* < 0.01; step 4 = 1.37 *p* < 0.01	Significant Diff
13	Jafari et al. 2020 [52]	*n* = 62	Not stated	Not stated	1. Self-efficacy (medians values provided for 15 questions). Frequency of practice behaviours (medians values provided for 11 questions)	*n* = 62	Not stated	Not stated	1. Self-efficacy (medians for 15 questions): *p* < 0.05. Frequency of practice behaviours (medians for 11 questions): 8 of 11 questions *p* < 0.05	Self-efficacy significantly increased across all 15 competencies (*p* < 0.05). Frequency of 8 out of 11 practice behaviours increased significantly (*p* < 0.05)	Significant Diff
14	Jedwab et al. 2022 [53]	*p* = 550	37.89 mean	M = 47 F = 491 Other = 8 Missing = 4	1. Well-being: well-being index = 64.00. Maslach Burnout: exhaustio*n* = 1.67, cynicism = 1.33, reduced efficiency = 1.67. 2. Work engagement: Satisfactio*n* = 7.81. Intention to stay = 8.10. Utrecht Work Engagement Scale—vigour = 3.40, dedicatio*n* = 4.30, absorptio*n* = 4.24. Career trajectory satisfactio*n* = 3.65. Perceived psychological safety = 2.91. Motivation to use technology: perceived confidence = 3.36, perceived external drivers = 0.02	*n* = 392	39.36 Mean	M = 32 F = 352 Other = 6 Missing = 2	1. Well-being: well-being index = 56.00. Maslach Burnout: exhaustio*n* = 2.00, cynicism = 1.33, reduced efficiency = 2.33. 2. Work engagement: Satisfaction 6.99. Intention to stay = 7.53. Utrecht Work Engagement Scale—vigour = 3.03, dedicatio*n* = 3.98, absorptio*n* = 4.12. Career trajectory satisfactio*n* = 3.34. Perceived psychological safety = 2.98. Motivation to use technology: perceived confidence = 3.57, perceived external drivers = − 0.23	Work satisfaction (r = 0.23, *p* ≤ 0.001), intention to stay (r = 0.11, *p* = 0.001) and well-being (r = 0.17, *p* ≤ 0.001) decreased, perceived competence increased (r = 0.10, *p* = 0.002) despite decreased autonomy (r = 0.10, *p* = 0.003). Two of three dimensions of work engagement decreased (vigour r = 0.13, *p* ≤ 0.001; dedication r = 0.13, *p* ≤ 0.001), all burnout dimensions increased (exhaustion r = 0.08, *p* = 0.012, cynicism r = 0.07, *p* = 0.04 and reduced efficiency r = 0.32, *p* ≤ 0.001). More burnout symptoms reported (95% CI 4.6–4.7%, *p* = 0.036), were less engaged (95% CI 49.6–49.9%, *p* ≤ 0.001) and career trajectory satisfaction decreased (r = 0.15, *p* ≤ 0.001)	Significant Diff
15	Johnston et al. 2020 [54]	*n* = 144 (at entry to medical program) NOTE: only *n* = 144 completed pre and post data	Cannot determine *n* = 144 as data for all entry surveys *n* = 3851 combined	Cannot determine *n* = 144 as data for all entry surveys *n* = 3851 combined	Intention to practice in rural and other underserved areas: *n* = 144 individual entry data not presented	*n* = 144 (at exit from medical program)	Cannot determine *n* = 144 as data for all exiting surveys *n* = 1187 combined	Cannot determine *n* = 144 as data for exiting surveys *n* = 1187 combined	Intention to practice in rural and other underserved areas: *n* = 144 individual exit data not presented	No significant change in proportion of learners intending to practice in rural areas *p* = 0.644	Non-significant Diff
16	Martin et al. 2019 [55]	*n* = 787	Not stated	Not stated	Confidence in skills and knowledge (pre-course): e.g., I have the necessary knowledge to help my clients with tobacco-related issues (51.7% agree);I am confident in my ability to address tobacco use (54.5% agree)	*n* = 765 (post) *n* = 416 (follow-up)	Not stated	Not stated	Confidence in skills and knowledge (post-course): e.g., I have the necessary knowledge to help my clients with tobacco-related issues (98.8% agree); I am confident in my ability to address tobacco use (98.6% agree)	Pre–post *p* < 0.001 for all confidence in skills and knowledge questions	Significant Diff
17	Mikolajczyk et al. 2021 [56]	*n* = 27	Not stated	M = 10 F = 17	Knowledge: mean percentage of MC questions × 12 correct = 55%	*n* = 59	Not stated	M = 25 F = 34	Knowledge: significant improvement in self-perceived knowledge across all CLD topics before the intervention cohort’s completion of residency	Knowledge: mean percentage of questions answered correctly by the third-year residents in the intervention cohort was 7.8 out of 12 (65%) compared to 6.8 out of 12 (55%) in the historic cohort (*p* = 0.04)	Significant Diff
18	Morshed et al. 2017 [57]	*n* = 201	Equally distributed 30–60 + years; less represented 20–29 years	M = 33 F = 168	NO Advanced degree (mean): 1. Skill = 6.61 2. Importance = 9.92 Advanced degree (mean): 1. Skill = 8.06 2. Importance = 10.11	*n* = 123	Equally distributed 30–60 + years; less represented 20–29 years	M = 18 F = 105	NO Advanced degree: 1. Skill = 7.40 2. Importance = 9.98 Advanced degree: 1. Skill = 8.03 2. Importance = 10.41	NO Advanced degree: 1. Skill *p* = 0.016 2. Importance *p* = 0.736 Advanced degree: 1. Skill *p* = 0.927 2. Importance *p* = 0.059	Non-significant Diff
19	Murthy et al. 2020 [58]	LF GROUP *n* = 107	not stated	M = 82 F = 25	1. CBE exam scores (mean): exam 1 = 5.77; exam 2 = 16.44; exam 3 = 23.79; exam 4 = 23.81	HF GROUP *n* = 107	Not stated	M = 77 F = 30	1. CBE exam scores (mean): exam 1 = 5.68; exam 2 = 17.30; exam 3 = 23.77; exam 4 = 23.92	Mean difference in exam scores between HF and LF models in exam 1 to 4 was not significantly different	Non-significant Diff
20	Neikrug et al. 2022 [59]	*n* = 251	Average = 44.7 years	M = 73 F = 177 Decline to state = 1	1. Knowledge = baseline to midpoint (mean difference = 7.6%, t = 10.6, *p* < 0.0001)	*n* = 251	Average = 44.7 years	M = 73 F = 177 Decline to state = 1	1. Knowledge = midpoint to post fellowship (mean difference = 4.23%, t = 5.59, *p* < 0.0001)	1. Knowledge = Repeated-measures analysis of the percent of correct answers on the knowledge score yielded significant improvement across the entire year (mean difference = 11.8%, t = 15.76, *p* < 0.0001)	Significant Diff
21	Ortega et al. 2018 [60]	Cohort 1 = 58 Cohort 2 = 111 Cohort 3 = 120 TOTAL = 289	*(Majority):* Cohort 1 = 72% 41–55 years Cohort 2 = 56% 41–55 years Cohort 3 = 50.8% 26–40 years	Cohort 1 = M = 3 F = 55 Cohort 2 = M = 13 F = 98 Cohort 3 = M = 9 F = 111 TOTAL = M = 25 F = 264	Learner performance pre-test/100%: Cohort 1 = (average) 65% Cohort 2 = 57.5% Cohort 3 = 53.4%	Cohort 1 = 48 Cohort 2 = 83 Cohort 3 = 89 TOTAL = 220	Not specific due to drop outs	Not specific due to drop outs	Learner performance post-test: Cohort 1 = 87% Cohort 2 = 89.3% Cohort 3 = 78.4% Mean total performance on modules: Cohort 1 = 95.4% Cohort 2 = 90.3% Cohort 3 = 89.6%	90% mean score on final exam NOTE: pre- and post-test data, therefore, were not individually matched; statistical significance could not be calculated	Non-significant Diff
22	Ortega et al. 2021 [61]	n-85	Not stated	Not stated	Only HuFSHI change scores provided (see difference between groups results)	*n* = 85	Not stated	Not stated	Only HuFSHI change scores provided (see difference between groups results)	Assessment and Mental State course:—t(7) = − 6.587, *p* < 0.000; Decision Making course: t(10) = − 4.411, *p* < 0.000; Place of Safety course: t(18) = − 4.932, *p* < 0.000; Crisis Resolution and Home Treatment Teams course: t(16) = − 4.737, *p* < 0.000	Significant Diff
23	Parmar et al. 2022 [62]	*n* = 161	Average 37 years	M = 59 F = 101	Knowledge and confidence = out of a score of 50 mean score 38.90	*n* = 161	Average 37 years	M = 59 F = 101	Knowledge and confidence = out of a score of 50 mean score 46.60	Post education scores significantly higher than pre *P* < 0.0001	Significant Diff
24	Playford et al. 2020 [63]	*n* = 776 (end of placement measure) as a student M = 124 F = 624; *n* = 474 (1 year post-graduation)—*results presented are for n* = *474*	Not stated	M = 77 F = 393 (1 year follow-up)	Location of practice—urban or rural = 26% in rural practice; rural background had the strongest relationship with early rural practice	*n* = 244 (15–17 year post-graduation)	Not stated	M = 77 F = 161 (15 –17 years follow-up)	Location of practice—urban or rural = most were practising in RA 1 (major cities) locations (193/240), with the remainder in (rural areas) RA 2 (23/240), RA 3 (19/240) and RA 4–5 (5/240). This gave a total of 47/240 (20%) practising rurally	Significant association between region practising 1 year post-graduation and region practising 15–17 year post-graduation (*p* < 0.001); significantly associated with long term rural practice were location of first job (*p* < 0.001) and rural background (*p* < 0.007)	Significant Diff
25	Risendal et al. 2022 [64]	*n* = 254	Not stated	M = 34 F = 220	Knowledge: percent of correct responses = 25%	*n* = 218	Not stated	M = 29 F = 189	Knowledge: Percent of correct responses = 46%	Knowledge: Percent of correct answers overall pre- to post-test *p* ≤ 0.0001. 14/15 were significant	Significant Diff
26	Salehi et al. 2021 [65]	*n* = 330 (for knowledge) (*n* = 293 for Confidence) (*n* = 74 for OSCE)	31.3 (mean)	M = 80 F = 250	Knowledge = 52% ± 11.2 Clinical skills = majority not competent at baseline (66% in physical assessment and 52% in communication)	*n* = 330 (for knowledge) (*n* = 293 for Confidence) (*n* = 74 for OSCE)	31.3 (mean)	M = 80 F = 250	Knowledge = 71% ± 9.2 Clinical skills competency: 96% in physical assessment, 99% in communication, and 100% in emergency	Knowledge = 37% increase, *P* = 0.000 Clinical skills 14 month follow-u*p* = Physical Assessment 3.7 ± 0.4 (*p* = 0.1); Communication 3.5 ± 0.4 (*p* = 0.000); Emergency 3.4 ± 0.6 (*p* = 0.000)	Significant Diff
27	Sibrian et al. 2022 [66]	*n* = 50	Not stated	Not stated	Not stated. Nil results presented	*n* = 50	Not stated	Not stated	Numbers not provided	Not stated	Non-significant Diff
28	Tran et al. 2019 [67]	*n* = 86	Not stated	M = 6 F = 80	1. Leadershi*p* = 4.8 (mean) 2. Management = 3.8 (mean) 3. APN Specific = 4.1 (mean)	*n* = 86	Not stated	M = 6 F = 80	1. Leadershi*p* = 6.1 (mean) 2. Management = 5.6 (mean) 3. APN Specific = 5.8 (mean)	Mean scores *p* < 0.001	Significant Diff
29	Vesel et al. 2015 [68]	*n* = 129	Not stated	Not stated	1. Coping skills = average 2.79 2. Perceived stress levels (only post-test) = average 2.48	*n* = 157	Not stated	Not stated	1. Coping skills = positive and significant diffs from retrospective to post test (*p* = 0.000); pre = average 2.63, post = 3.23 2. Perceived stress levels (only post-test) average = 2.40	Overall Coping = On average higher coping strategy levels in intervention group (score of 3.23) vs comparison (2.79)—Significant diff (*p* = 0.000). On Average = lower stress levels in intervention group (score of 2.40) vs comparison (2.48)—Significant diff (*p* = 0.034)	Significant Diff
30	Zhang et al. 2021 [69]	*n* = 47	mean 38.3	M = 33 F = 14	(Perceived Stress Scale (PSS)-10, Generalised Anxiety Disorder (GAD)-7) PRE-COVID: 1. Medical service statistics—Number of OP’s = 41.9 ± 11.9; LOS = 0.4 ± 1.0. 2. Workload—weekly working hours 1884.9 ± 34.1; 3. PSS = PSS-10: 4.3 ± 2.4; 4. GAD = 4.0 ± 2.3	*n* = 47	Mean 38.3	M = 33 F = 14	AFTER COVID OUTBREAK: 1. Medical service statistics—Number of OP’s = 37.6 ± 11.8 per week; LOS = 3.1 ± 3.9 days; 2. Workload—weekly working hours 2023.5 ± 67.3 h; 3. PSS = PSS-10: 7.5 ± 3.9; 4. GAD = 9.4 ± 4.0	AFTER COVID OUTBREAK: 1. Medical service statistics—*p* = 0.49; LOS = *p* = 0.02; 2. Workload—weekly working hours *p* < 0.001; 3. PSS = *p* < 0.001; 4. GAD = *p* < 0.001	Significant Diff

PEDro scores for risk of bias ranged from 1 to 6 out of 10, with an average of 5 out of 10 (Appendix 2). Only two studies used randomisation [49, 58] and none reported blinding of participants or assessors, or allocation concealment. Most investigations reported on the remaining criteria. Study quality was comparatively low with 23/30 of the studies receiving a weak rating on the Quality Assessment tool for Quantitative Studies, with the remaining receiving a moderate rating (Appendix 3). Studies were most often weak for blinding (*n* = 23), cofounders (*n* = 18), and data collection methods (*n* = 16).

While the included articles often reported multiple domains that the intervention aimed to influence, the following paragraphs are based on the primary outcome domains.

### Education

For the 24 studies with a primary focus on a care workforce education program, there were marked variations in the education interventions provided (e.g., leadership development, condition-based education programs, extending scope of practice), the location (e.g., USA, Australia, East Africa), the setting (e.g., hospitals, aged care, primary care), and the outcome measures used. As shown in Tables 1 and 2, both validated and unvalidated measurement tools were to measures the change in workforce knowledge [40–46, 48–52, 55–62, 64–67]. Of the 24 studies, only two used a randomised controlled trial design, [49, 58] with the rest using a pre–post study design. Only four did not report a significant improvement in staff knowledge. The education included topics, such as cancer education [56], obesity management, [57] simulation training for a breast examination, [58] leadership development, [67], and clinical nursing skills [66].

Of the six studies that included consumer engagement and co-design, all reported benefits. These investigations reported significant improvements in staff knowledge relating to topics, such as electronic health record implementation, [50] dementia care, [51] smoking cessation, [55] care-giver centred care, [62] cancer survivorship, [64] and paediatric nursing [65]. Of the five education studies that focused on the rural workforce, all reported a significant improvements. This pertained to staff knowledge related to midwifery, [41] non-physician extended scope of practice, [49] leadership development, [67] smoking cessation, [55] and cancer survivorship [64].

Of the 24 education investigations, 18 were conducted in high income countries, [40, 43–46, 50–52, 55–57, 59–61, 64, 66, 67] and six were conducted in low-to-low–middle income countries, [41, 42, 48, 49, 58, 65]. Of the four studies that did not demonstrate significant findings, three were based on the USA (high income country) and aimed to improve cancer knowledge, [57] nursing leadership, [60], and care workforce needs during COVID-19 [66]. One was based on Rwanda and focussed on low and high fidelity medical education for clinical breast examination. Both groups showed improvements in knowledge, yet there were no between group differences [58].

### Recruitment and retention

Two studies reported on interventions that focused on rural workforce recruitment and retention, and both were conducted in Australia (high income country) [20, 63]. The first was on the Training for Health Equity Network (THEnet) to improve staff recruitment into the rural medical workforce. This did not report an increase in the proportion of learners intending to practice in rural areas [54]. The study was directed towards allied health and nursing students completing a rural placement in their final year of study [63]. The authors reported a significant association between the number of weeks of rural placement in the final year of study, and initial rural recruitment. However, the significant association reported for recruitment was not maintained for retention 15–17 years later [63].

### Safety

Three investigations reported on safety relating to staff mental health and well-being [53, 68, 69]. These showed a significant improvement in primary outcomes. These included an increase in well-being and satisfaction following the introduction of the electronic medical record for the nursing workforce in Australia (high income country), [53] an increase in coping skills following the Helping Health Workers Cope program for the rural health and nursing workforce in West Africa (Sierra Leone; low income country), [68] and a reduction in perceived stress following the introduction of a triage and disinfection protocol for the medical, nursing and allied health workforce in South Sudan (low income country) [69]. There was an additional study the focussed on minimum nurse-to-patient ratios with the rational of improving patient safety. While this reported a significant improvement in the nurse to patient ratios following the introduction of staffing mandates, it did not specifically report the impact on patient safety (conducted in USA; high income country) [47].

Four effective evidence-based strategies from this rapid review have been established to add to the current literature base and improve professional workforce recruitment, retention, safety, and education. Where appropriate, it has been noted when the strategy is aligned to the WHO guideline on health workforce development, attraction, recruitment and retention in rural and remote areas [19].

### Strategy 1 (recruitment and retention)

To support long term retention, implement strategies to recruit early career staff, especially to rural locations.

- ***New evidence from this rapid review:*** As the location of nursing and allied health practice in the first year post-graduation is a significant predictor for retention and the location of practice 15 + year post-graduation, there is a need to implement strategies to recruit professionals to rural locations, especially in the first year of practice.
- ***Alignment to WHO guidelines:*** “WHO recommends using targeted admission policies to enrol students with a rural background in health worker education programmes” [19] and “WHO recommends exposing students of a wide array of health worker disciplines to rural and remote communities and rural clinical practices” [19]

### Strategy 2 (safety)

To support health professional mental and physical well-being, implement strategies for workload management alongside safety training and psychological support.

- ***New evidence from this rapid review:*** Optimising workload management can improve health professional health and well-being. Examples include an effective triage process [69], workload management using streamlined electronic medical records [53], and implementing staff to client ratio mandates [47].
- ***Alignment to WHO guidelines***: “WHO recommends ensuring a safe and secure working environment for health workers …” [19]

### Strategy 3 (education)

To maximise learning, ensure that health professionals have access to contextually relevant and ongoing professional development to improve capabilities and professional knowledge.

- ***New evidence from this rapid review:*** Contextually relevant professional education and development improves staff retention by focussing on staff needs, interventions relevant to the care setting, the patient population, cultural considerations, as well as providing evaluations of the impact of the new knowledge and skills.
- Aligned to WHO guidelines: “WHO recommends designing and enabling access to continuing education and professional development programmes that meet the needs of … workers to support their retention …” [19]

### Strategy 4 (align recruitment and retention strategies to workforce categories)

There is a need to differentiate recruitment, retention and education strategies for different professional health and care workforce categories as needs vary.

- ***New evidence from this rapid review:*** Contextually relevant education, training and support needs to be matched to specific requirements of different professions, such as nursing, allied health and medicine. Non-registered, non-credentialed care workers may have different learning needs and interventions need to be tailored accordingly [70].

## Discussion

From this rapid review, four new workforce strategies emerged; early career rural recruitment supports rural retention; workload management is essential for workforce well-being; learning must be contextually relevant; and there is a need to differentiate recruitment, retention and education strategies for different professional health and care workforce categories because needs vary. The care economy is one of the most rapidly growing sectors in the world and significant workforce shortages are predicted [3, 16]. The International Centre on Nurse Migration (2022), recommends the implementation of national and international action plans to improve care workforce recruitment and retention, supported by high-quality, large-scale research trials [71]. Trials are needed to measure the impact and outcomes of interventions to address issues, such as workforce demand–supply gaps, staff burnout and how to enhance work satisfaction. A recurrent theme in the articles reviewed was that staff education is a powerful determinant of these elements. Education was evaluated in most trials and other interventions included leadership training, mental health support for workers and training in the use of new technologies to support care delivery. Digital innovations, care delivery simulations, and implementation of electronic medical records improved worker satisfaction. Another theme was the need to establish a considered, co-ordinated, responsive, co-designed approach to support the care workforce and to maximise workforce recruitment, retention, safety, career progression and knowledge.

Consistent with Randell et al. (2021) [72], no global investigations were identified that provided a world-wide approach to coordinated workforce recruitment, retention, and enhancement. Each of the studies reviewed was site-specific and directed towards local needs and priorities. There was no clear pattern as to the impact of the economic status of the different countries on care workforce recruitment, retention, knowledge or safety. Many of the trials had similar designs and findings, yet they lacked the scale or reach across care economy domains to have a sustained impact nationally or globally. Of concern, most were of low methodological quality and only a few were of moderate quality. Our review also highlighted minimal involvement of consumers of health and social care services in the co-design of research or services. The economic evaluations of care workforce interventions were not reported.

A previous systematic review [4] showed low poor levels of care worker recruitment, as well as burnout and high staff turnover in the child welfare sector. These problems were related to personal factors, such as low levels of commitment to welfare, as well as emotional exhaustion in some people, and organisational factors, such as poor supervision and low co-worker support. Low salaries and benefits were also important elements that influenced decisions by child welfare workers to stay in the field. The emotional labour of working with people with poor health and other distressing circumstances was associated with fatigue and burnout. Job-related stress was associated with high workloads, combined with time pressures and ambiguous roles [73]. The current review also reiterated that most research has been focused on health, with social care largely overlooked, despite indications of increasing demands including global demographic trends projecting reduced availability of informal carers and growing need for long-term care for elderly people [74–76].

Our findings are congruent with Johnston’s rapid review on staff recruitment, retention, and development in the social care domain [77]. In addition the results align with the Australian National Care and Support Workforce Strategy (2022) [3] which identified five principles to support a strong workforce: target migration, activate and coordinate industry, remove barriers, skill up workers and use data to drive change. Across the care economy there is a need to attract people from diverse backgrounds, including migrants, youth and older women returning to the workforce, to meet the growing needs of diverse populations. Education and training of staff is central to retention, as is designing safe work environments and enabling attractive career pathways, supported by programs that include care provider well-being. A recent practical inquiry about attracting young people, particularly young indigenous people, into care work found that strategies of engagement were central and essential to interest them. There was also a need to identify specific care roles for which they were suited, preparing them for those roles and retaining them in that workforce [78].

Our review indicated that industry could play a pivotal role in removing barriers to care worker recruitment, retention, education and safety. The actions of care organisations and companies are influenced by key legislative drivers and those operating in the care economy sector are not immune. In the UK and Australia legislation has been adopted to combat forced labour and uphold decent working conditions. The Modern Slavery Act (2018) [79] requires companies with annual turnover in excess of $100m to report against risks in their supply chains and operations that signify significant risks in the employment of workers. Moreover, the United Nations Sustainable Development Goals (Sustainable Development Goals, 2021 [80]) of which Australia and many countries are signatories, pertain to decent work, economic growth, full and productive employment and equal pay for work of equal value. Companies and organisations are increasingly reporting on labour issues, including discrimination, human resource management, working conditions, industrial relations, and occupational health and safety [81]. Even through organisations and companies operating in the care economy arguably have less normative and regulatory pressures to adopt such practices compared to those operating in high-risk settings, such as mining and energy, the advent of legislation may increase such pressures across the sector. As the health and social care sectors are finding themselves under pressure to recruit qualified workers, recruitment and retention are likely to be influenced by how company actions are perceived by potential workers. Workers have many options for employment due to shortage of labour that followed the impact of COVID-19 restrictions, and they expect their employers to abide by legislation, and to act ethically in terms of their employment practices. Health and social care organisations are increasingly recognising the strong link between care worker well-being and safe and high-quality services [82].

When reviewing the literature, it became clear that specific strategies are needed for different health and care categories. The current manuscript focused on professional, qualified care workforces, such as nurses, doctors and allied health professionals, who have already spent many years in education and training to prepare them for their roles. Other members of the care workforce such as peer support workers, volunteers, personal care attendants, allied health assistants and nursing assistants have different needs for training in the workplace [70, 83, 84]. For example, over 50% of personal care attendants and 37% of aged care and disability workers are non-English speaking migrants [85]. There is emerging evidence of poor job quality for this cohort of workers, with a survey of 16,000 residential and community care workforce reporting predominantly casual status and underemployment [86]. Education, training, policies and systems need to take into account the needs of different workforce sectors, as recommended by recent reports [87, 88].

There are several limitations of this rapid review. The focus was over 8 recent years, and relevant studies outside of this time period were excluded. The search strategy also excluded studies published in languages other than English, and may have overlooked meaningful cultural contexts [89]. We also excluded qualitative studies, which can provide data to better understand the experiences of care workers and organisations. Future studies need to include a sector specific analysis of care workforce needs and recommendations.

## Conclusion

With the growing importance of the care workforce and predicted long term global shortages exacerbated by ageing populations, evidence-based strategies to recruit and retain workers are vital. The growing and increasingly diverse workforce within the care economy requires attention to improve the quality of care for consumers and the service systems they access. Efforts to support the well-being and retention of care workers need to include the voice and lived experience of consumers, be sustainable and based on evidence. Recruiting a more diverse workforce, ensuring worker well-being and safety, and providing education and career development are essential to meet the current and future needs of the care economy.

## Data Availability

The data sets used and/or analysed during the current study are available from the corresponding author on reasonable request.

## Appendix 1: example of rapid review search strategy for Ovid MEDLINE

**Table:**

	Query	Results from 15 Nov 2022
1	Care Workforce.mp	1560
2	Health Workforce.mp	16,950
3	Ageing Workforce.mp	94
4	Aged Care Workforce.mp	56
5	Disability Workforce.mp	11
6	Rehabilitation Workforce.mp	38
7	Nursing Workforce.mp	2054
8	Allied Health Workforce.mp	62
9	Medical Workforce.mp	699
10	Mental Health Workforce.mp	209
11	Social Workforce.mp	4
12	Housing Workforce.mp	0
13	Homelessness Workforce.mp	0
14	Childcare Workforce.mp	0
15	Child Protection Workforce.mp	1
16	Family Violence Workforce.mp	1
17	Domestic Violence Workforce.mp	0
18	Family Service* Workforce.mp	1
19	Rural Workforce.mp	205
20	Remote Workforce.mp	18
21	Indigenous Workforce.mp	14
22	Hospital Workforce.mp	73
23	Home Care Workforce.mp	31
24	Community Workforce.mp	20
25	Drug Workforce.mp	6
26	Alcohol Workforce.mp	2
27	1 or 2 or 3 or 4 or 5 or 6 or 7 or 8 or 9 or 10 or 11 or 12 or 13 or 14 or 15 or 16 or 17 or 18 or 19 or 20 or 21 or 22 or 23 or 24 or 25 or 26	21,027
28	Recruit*.mp	402,763
29	Retention.mp	187,007
30	Safe*.mp	964,726
31	Educat*.mp	1,067,939
32	28 or 29 or 30 or 31	2,489,424
33	27 and 32	8997
34	limit 33 to English language	8449
35	limit 34 to year = "2015 -Current"	3420

## Appendix 2: Scores for the PEDro [37, 38] risk of bias assessment

**Table:**

	PEDro: risk of Bias (Yes = 1; No = 0)										
Included paper	Random allocation of participants to groups	Allocation concealment	Similarity of groups at baseline regarding the most important prognostic indicators	Blinding of all participants	Blinding of all therapists who administered the therapy or intervention	Blinding of all assessors who measured at least one key outcome	Measures of at least one key outcome were obtained from more than 85% of the participants initially allocated to groups	All participants for whom outcome measures were available received the treatment or control condition as allocated or, where this was not the case, data for at least one key outcome was analysed by “intention to treat”	The results of between-group statistical comparisons are reported for at least one key outcome	The study provides both point measures and measures of variability for at least one key outcome	Score out of 10
Abdulla et al. 2020	0	0	1	0	0	0	1	1	1	1	5
Alwy Al-Beity et al. 2020	0	0	1	0	0	0	1	1	1	1	5
Ayisi-Boateng et al. 2022	0	0	1	0	0	0	1	1	1	1	5
Azoulay et al. 2021	0	0	1	0	0	0	1	1	1	1	5
Bennett et al. 2022	0	0	1	0	0	0	0	1	1	1	4
Chicoine 2022	0	0	1	0	0	0	1	1	1	1	5
Clancy et al. 2020	0	0	1	0	0	0	1	1	1	1	5
Dierkes et al. 2022	0	0	1	0	0	0	1	1	1	1	5
Downing et al. 2016	0	0	1	0	0	0	1	1	1	1	5
Gajewski et al. 2019	1	0	1	0	0	0	1	1	1	1	6
Gordon et al. 2022	0	0	1	0	0	0	1	1	1	1	5
Islam et al. 2020	0	0	1	0	0	0	1	1	1	1	5
Jafari et al. 2020	0	0	1	0	0	0	1	1	1	1	5
Jedwab et al. 2022	0	0	1	0	0	0	1	1	1	1	5
Johnston et al. 2020	0	0	0	0	0	0	0	1	1	0	2
Martin et al. 2019	0	0	1	0	0	0	1	1	1	1	5
Mikolajczyk et al. 2021	0	0	1	0	0	0	1	1	1	1	5
Morshed et al. 2017	0	0	0	0	0	0	1	1	1	1	4
Murthy et al. 2020	1	0	1	0	0	0	1	1	1	1	6
Neikrug et al. 2022	0	0	1	0	0	0	1	1	1	1	5
Ortega et al. 2018	0	0	1	0	0	0	1	1	0	1	4
Ortega et al. 2021	0	0	1	0	0	0	1	1	1	0	4
Parmar et al. 2022	0	0	1	0	0	0	1	1	1	1	5
Playford et al. 2020	0	0	1	0	0	0	1	1	1	1	5
Risendal et al. 2022	0	0	1	0	0	0	1	1	1	1	5
Salehi et al. 2021	0	0	1	0	0	0	1	1	1	1	5
Sibrian et al. 2022	0	0	1	0	0	0	0	0	0	0	1
Tran et al. 2019	0	0	1	0	0	0	1	1	1	1	5
Vesel et al. 2015	0	0	1	0	0	0	1	1	1	1	5
Zhang et al. 2021	0	0	1	0	0	0	1	1	1	1	5

## Appendix 3: Individual scores for the quality assessment tool for quantitative studies [90]

**Table:**

Quality assessment tool for quantitative studies: quality assessment														
Included paper	Selection Bias (S = Strong; M = Moderate; W = Weak)	Study Design (S = Strong; M = Moderate; W = Weak)	Confounders (S = Strong; M = Moderate; W = Weak)	Blinding (S = Strong; M = Moderate; W = Weak)	Data Collection Methods (S = Strong; M = Moderate; W = Weak)	Withdrawals and Drop-outs (S = Strong; M = Moderate; W = Weak)	Intervention Integrity: received allocated intervention/exposure	Intervention Integrity: consistency of intervention measured	Intervention Integrity: likelihood of contamination	Analysis: unit of allocation	Analysis: unit of analysis	Analysis: appropriate statistical analysis	Analysis: is analysis via intention to treat	Global rating
Abdulla et al. 2020	S	M	M	M	W	S	80–100%	Can’t tell	No	Individual	Individual	Yes	No	M
Alwy Al-Beity et al. 2020	M	M	M	W	S	S	80–100%	Can’t tell	No	Individual	Individual	Yes	No	M
Ayisi-Boateng et al. 2022	W	W	W	W	S	N/A	80–100%	Can’t tell	No	Practice	Individual	Yes	Can’t tell	W
Azoulay et al. 2021	W	M	W	M	S	N/A	80–100%	Can’t tell	Can’t tell	Individual	Individual	Yes	No	W
Bennett et al. 2022	M	S	S	W	S	S	80–100%	Can’t tell	Can’t tell	Community	Community	Yes	Can’t tell	M
Chicoine 2022	W	M	W	W	M	W	< 60%	Can’t tell	Can’t tell	Organisation	Individual	Yes	No	W
Clancy et al. 2020	S	S	M	W	S	S	80–100%	Can’t tell	No	Organisation	Organisation	Yes	Can’t tell	M
Dierkes et al. 2022	S	S	M	W	S	N/A	80–100%	Yes	No	Organisation	Organisation	Yes	Can’t tell	M
Downing et al. 2016	W	M	W	W	W	S	80–100%	Can’t tell	No	Organisation	Individual	Can’t tell	No	W
Gajewski et al. 2019	M	M	M	M	W	W	80–100%	Can’t tell	Can’t tell	Organisation	Organisation	No	Can’t tell	W
Gordon et al. 2022	W	W	W	W	M	W	Can’t tell	Can’t tell	Can’t tell	Individual	Individual	Yes	Can’t tell	W
Islam et al. 2020	M	W	W	W	W	W	80–100%	Can’t tell	No	Individual	Individual	Yes	Can’t tell	W
Jafari et al. 2020	M	W	W	W	W	M	60–79%	Can’t tell	No	Individual	Individual	Yes	Can’t tell	W
Jedwab et al. 2022	W	M	W	M	M	M	< 60%	Can’t tell	Can’t tell	Organisation	Organisation	Yes	Can’t tell	W
Johnston et al. 2020	M	M	M	W	M	M	60–79%	Can’t tell	Can’t tell	Organisation	Organisation	Yes	Can’t tell	M
Martin et al. 2019	M	M	W	W	W	W	Can’t tell	Yes	Can’t tell	Individual	Individual	Yes	Can’t tell	W
Mikolajczyk et al. 2021	M	M	W	W	W	S	80–100%	Can’t tell	No	Individual	Individual	Yes	Yes	W
Morshed et al. 2017	W	W	M	W	W	S	Can’t tell	Can’t tell	Can’t tell	Individual	Individual	Yes	No	W
Murthy et al. 2020	S	S	S	M	W	S	80–100%	Yes	No	Individual	Individual	Yes	Yes	M
Neikrug et al. 2022	W	M	S	W	S	W	80–100%	Yes	No	Individual	Individual	Yes	Yes	W
Ortega et al. 2018	W	W	W	W	W	W	Can’t tell	Can’t tell	Can’t tell	Community	Community	Yes	No	W
Ortega et al. 2021	M	M	W	W	W	N/A	Can’t tell	Can’t tell	Can’t tell	Community	Community	Yes	Can’t tell	W
Parmar et al. 2022	S	M	W	W	S	S	80–100%	Yes	Can’t tell	Individual	Individual	Yes	No	W
Playford et al. 2020	W	M	W	W	S	W	80–100%	Yes	Can’t tell	Individual	Individual	Yes	No	W
Risendal et al. 2022	M	W	M	W	W	W	60–79%	Can’t tell	No	Organisation	Individual	Yes	No	W
Salehi et al. 2021	W	M	W	M	W	S	Can’t tell	Yes	Yes	Organisation	Organisation	Yes	Can’t tell	W
Sibrian et al. 2022	W	M	W	M	W	W	Can’t tell	Can’t tell	Can’t tell	Organisation	Organisation	Can’t tell	Can’t tell	W
Tran et al. 2019	M	M	M	W	W	M	< 60%	No	No	Organisation	Organisation	Yes	Can’t tell	W
Vesel et al. 2015	M	M	W	W	S	M	80–100%	Can’t tell	Can’t tell	Organisation	Organisation	No	No	W
Zhang et al. 2021	M	W	W	W	W	W	Can’t tell	No	No	Practice	Practice	Yes	Can’t tell	W
